# Environmental and Structural Factors Associated with Bacterial Diversity in Household Dust Across the Arizona-Sonora Border

**DOI:** 10.21203/rs.3.rs-3325336/v1

**Published:** 2023-09-29

**Authors:** Lauren Benton, Nicolas Lopez-Galvez, Chloe Herman, Greg Caporaso, Emily Cope, Cecilia Rosales, Mercedes Gameros, Nathan Lothrop, Fernando Martínez, Anne Wright, Tara Carr, Paloma Beamer

**Affiliations:** University of Arizona; University of Arizona; Northern Arizona University; Northern Arizona University; Northern Arizona University; University of Arizona; University of Arizona; University of Arizona; University of Arizona; University of Arizona; University of Arizona; University of Arizona

## Abstract

We previously reported that asthma prevalence was higher in the United States (US) compared to Mexico (MX) (25.8% vs 8.4%). This investigation assessed differences in microbial dust composition in relation to demographic and housing characteristics on both sides of the US-MX Border. Forty homes were recruited in the US and MX. Home visits collected floor dust and documented occupants’ demographics, asthma prevalence, and housing structure and use characteristics. US households were more likely to have inhabitants who reported asthma when compared with MX households (30% vs 5%) and had significantly different flooring types. The percentage of households on paved roads, with flushing toilets, with piped water and with air conditioning was higher in the US, while dust load was higher in MX. Significant differences exist between countries in the microbial composition of the floor dust. Dust from US homes was enriched with *Geodermatophilus*, whereas dust from Mexican homes was enriched with *Alishewanella* and *Chryseomicrobium*. A predictive metagenomics analysis identified 68 significantly differentially abundant functional pathways between US and MX. This study documented multiple structural, environmental, and demographic differences between homes in the US and MX that may contribute to significantly different microbial composition of dust observed in these two countries.

## Introduction

The indoor household environment, where people spend up to 90% of their time, contains a complex ecosystem of organisms, including trillions of microbes [1–3]. Some of these microorganisms adversely affect human health, while others can have a positive health impact [4–6]. Previous studies have demonstrated that indoor microbiome diversity can play an important role in the protection against immune-mediated diseases [7]. For example, there are microbial diversity differences in dust samples from houses of children without asthma versus children with asthma [8, 9]. Urbanization has led to decreasing microbial diversity in people’s homes [10]. One proposed premise for the difference in microbiome diversity is that urbanization can result in more enclosed built environments with limited exposure to diverse microbiota [10–13]. Additionally, anthropogenic pollutants present in urban environments, along with high use of household disinfectants present in Western modern lifestyles, may alter the microbial balance in household environments by potentially reducing its biodiversity [14–16]. The microbial community in household environments can be linked to the number of occupants, both human and pets. Also, it has been proposed that house characteristics such as the design, humidity, type of ventilation, and the geographical location of the house could play an important role in shaping the microbial community [3, 4, 17]. Geography, however, may be a stronger driver of building environment microbiome composition than other factors such as building material [18].

Regions that are similar in climate and population genetics but have different living conditions can illustrate the impact of varying household exposures on human health. For example, Finnish and Russian Karelia are two bordering regions that developed differently after World War II. Russian Karelia continued small scale farming and is, overall, a more rural environment, whereas Finnish Karelia went through rapid economic growth and urbanization. These areas also have different rates of atopic disease, proposed to be related to differences in drinking water microbes and other exposures [19–21]. Individuals living in Russian Karelia have greater microbial biodiversity present in the nasal passage and skin compared to Finnish Karelia [20].Analogous environmental differences can be observed between East and West Germany, as well as between Amish and Hutterite communities [22, 23]. After the Berlin Wall fell, East and West Germany were very different, with West Germany being more modernized and wealthier; this led to West Germany having higher rates of allergy and asthma [23, 24]. Children who grow up on traditional European farms have less prevalence of asthma, and this effect may be in part due to exposure to a greater diversity of microbes in early life [22, 25, 26]. The prevalence of asthma is much greater in Hutterite communities compared to Amish communities even though they share similar genetic ancestry and live in similar environments. This is thought to be due to differences in farming practices, as the populations have similar genetic ancestry. The Amish communities practice more traditional farming, whereas the Hutterite use modern farming techniques [22]. Together, we see that evaluating areas with similar geographic, climatic, and genetic background of inhabitants allows us to examine how socioeconomic, land use, and environmental determinants contribute to health disparities, such as the development of chronic diseases like asthma.

Another striking example of political and economic divide impacting similar ethnic and geographic areas can be observed along the US-Mexico Border. Nogales, Arizona, US, and Nogales, Sonora, MX are sister cities along the US-Mexico Border, that together are called Ambos Nogales (i.e., Both Nogales). These cities were once united with shared cultures, families, and economies, but due to changing political pressures, became divided, similar to East and West Berlin. Nogales, MX is the larger of the two cities, with a population of 220,292 in 2010, compared to the 20,837 residents in Nogales, US. Tucson, Arizona, US is located only about 100 km north of Ambos Nogales, and although it has a much larger population (close to 1 million) it is much less densely populated (U.S. Census Bureau 2012; Gobierno Municipal Nogales Sonora 2010) There are measurable infrastructure differences between the US cities and Nogales, MX, as the cities located in the US have similar access to the US standards of sanitation, clean drinking water, and the American style of housing development. Within Nogales, MX, there are distinct neighborhoods with differing socioeconomic status (SES), which leads to different housing styles and variable access to municipal infrastructure. Some neighborhoods are similar to US neighborhoods with piped water and planned housing developments. Others, the *colonia marginadas*, or informal settlements, do not have access to municipal resources such as sanitation and drinking water [27, 28].

We previously found that there is four-fold higher prevalence of asthma amongst children of Mexican descent attending middle school in southern Tucson and Nogales, US compared to those attending middle school in Nogales, Sonora, MX, despite similar ethnic backgrounds and a limited geographic region [29]. We found the lowest rates of asthma at the school with lower socioeconomic status located in a *colonia marginada*. These findings lead to questions about whether there are differences in household microbial environments in the different cities. The objective of this study was to identify differences in housing characteristics, dust load, and microbial diversity in households located on both sides of the US-MX Border in Tucson, Arizona, US (TUS), Nogales, Arizona, US (NUS), and Nogales, Sonora, MX (NMX) that may contribute to asthma prevalence. This study also investigated two different neighborhoods within Nogales, MX, including a traditionally high SES neighborhood and low SES neighborhood.

## Results

### Study Population

Overall, 20 homes from TUS, 20 homes from NUS, and 40 homes from NMX (20 high SES and 20 low SES) were recruited into the study. The household and environmental characteristics were analyzed and compared between the countries (US vs MX) as well as between neighborhoods (TUS, NUS, high SES NMX, and low SES NMX). There was no significant difference in age or gender of the respondents between the US and MX, but the respondents in the US had significantly more years of education (Supplemental Table S1). US households had higher incomes and cleaned their floors less frequently (Table 1). The source of drinking water was also significantly different between homes in MX and homes in the US, with US homes utilizing public tap water (Table 1). US families were significantly more likely to have inhabitants with asthma than those in MX (Table 1). There were no significant differences in smoking, pets, or number of inhabitants between the US and high SES NMX, but households in low SES NMX had more inhabitants and fewer animals per household (Table 1).

### Household Characteristics

Although all cities were close to the US-MX Border and within 100 km of each other, there were striking differences between the homes by country. All the homes in high SES NMX had the same floor plan, as it was a planned subdivision. The percentage of homes with paved roads, flushing toilets, and piped water was significantly lower in low SES NMX (Table 2) compared with the other neighborhoods. The homes in the US differed from those in MX in type of structure (Table 2), with 33% of homes in US being apartments or trailers, while 100% of the homes in MX were detached or duplex houses (Table 2). Homes in the US had more rooms and more bathrooms than in MX. All homes in the US had either air conditioning, evaporative cooling, or both, whereas only a few of the homes in MX (10%) had either type of cooling (Table 2). Flooring also differed. Most of the rooms where the dust samples were collected in MX had smooth floors (93%), whereas 42% of samples in the US were collected from carpet or rugs (Table 2). Significantly more homes in TUS had mildew or moisture present than the other sites (Table 2).

### In-home floor dust loading

As presented in Table 3, we found significantly greater amounts of dust loading in MX homes (p=0.04) compared to US homes. In addition, the homes located on a dirt road had significantly higher (p=0.02) dust load than the homes located on an asphalt road. Significantly more dust was retrieved in homes with at least one pet (p=0.01), more than two children (p=0.01), and more than 4 residents (p=0.01) (Table 3). There were no significant differences in dust loading based on the number of rooms or number of adults living in the home. Finally, although we did not find any significant differences between the dust load by floor type in homes located in the US versus MX, there was a significantly higher (p=0.03) dust load within US homes that had some sort of carpet, in comparison to homes with smooth flooring (Supplemental Fig. S1). There were no differences in dust loading in relation to whether there were asthmatic inhabitants.

### Dust microbiome

**Figure 1.**
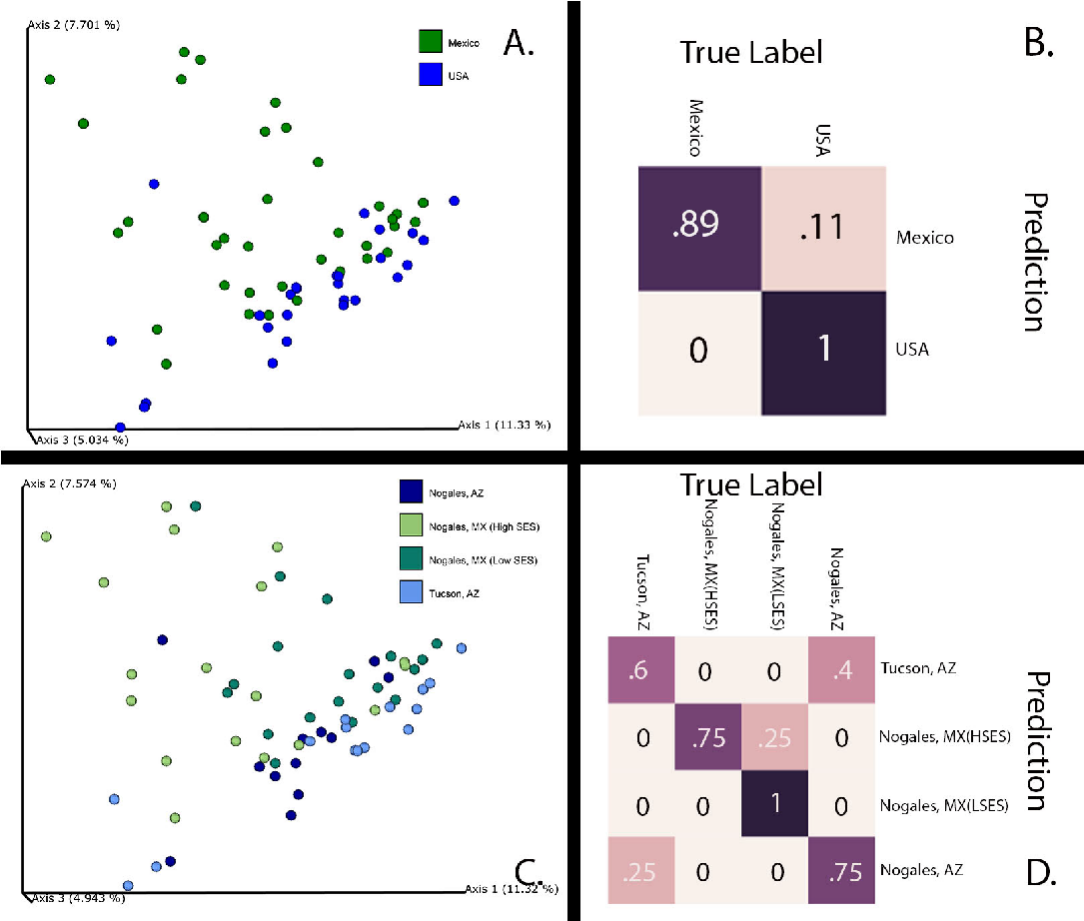
**A.** Unweighted UniFrac beta diversity plot comparing the United States of America (US) and Mexico (MX) house dust (PERMANOVA pseudo-F = 3.02 p < 0.001, q < 0.05). **B:** Random Forest classifier trained to differentiate the neighborhoods using the dust microbiome compositions. The classifier achieved an overall accuracy of 0.94. US samples were accurately labeled 100% of the time, and Mexican samples were accurately labeled 88% of the time. **C.**Unweighted UniFrac beta diversity plot comparing high SES NMX neighborhood vs low SES NMX, vs NUS vs TUS. **D.**Random Forest classifier trained to differentiate the neighborhoods using the dust microbiome compositions the model predicted NUS accurately 75% of the time; TUS 60% of the time: high SES NMX 75% of the time; and low SES NMX 100% of the time. LSES= low socioeconomic status Nogales, Sanora, Mexico; HSES= high socioeconomic status Nogales, Sanora, Mexico; MX=Mexico; USA= United States of America

**Figure 2:**
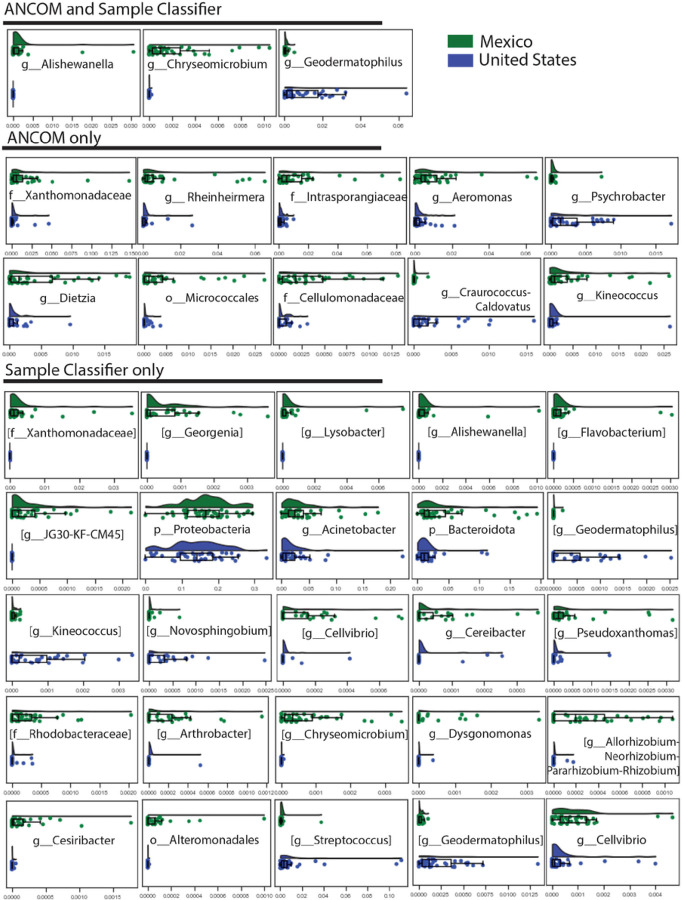
ANCOM and Sample Classifier comparing the house dust from homes in the United States vs Mexico. Top is Mexico and bottom is United States of America

The microbiome compositions, as measured by unweighted UniFrac, of the dust in the US samples and MX samples were significantly different prior to multiple comparisons correction, and nearly significant after multiple comparisons correction (PERMANOVA pseudo-F = 3.02 p < 0.001, q = 0.051; Fig. 1). We trained a random forest classifier to differentiate the neighborhoods using the dust microbiome compositions, and the classifier achieved an overall accuracy of 0.94, which was 1.9 times better than baseline accuracy (assigning the most common category to all samples; Fig. 1). US samples were accurately labeled 100% of the time, and MX samples were accurately labeled 88% of the time (Fig. 1). Our random forest model and ANCOM each reported many different genera, but both methods identified two of the same genera that were enriched in the MX dust (*A. Alishewanella,* and *Chryseomicrobium*)and one genus that was enriched in the US dust (*Geodermatophilus*) (Fig. 2).

The dust microbial composition between the four sites (two US cities and two MX neighborhoods) was significantly different (PERMANOVA pseudo-F = 2.33, p < 0.001; because all tests results reported in this paragraph were the same, we will report the results as F, p, and q-values for readability). The microbial composition in the dust from the higher SES neighborhood in NMX was significantly different from the US cities’ (NUS versus high SES NMX): F = 1.90, p = .002, q = 0.0024; TUS versus high SES NMX): F = 2.83, p < 0.001, q = 0.0015) and significantly different from low SES NMX: (F = 2.57, p < 0.001, q = 0.0015). The low SES NMX neighborhood dust microbiome composition was also significantly different compared to the US cites (NUS versus low SES NMX): F = 2.48, p < 0.001, q = 0.0015; TUS versus low SES NX: F = 2.74, p < 0.001, q = 0.0015) (Fig. 1). The US cities were not significantly different from each other (TUS versus NUS: F = 1.36, p = 0.071, q =0.0710 (Fig. 1). A sub-analysis showed that the house dust microbial composition differed when comparing the homes by road type (F = 2.59, p < 0.001, q < 0.001) and presence of air conditioning (air conditioning vs none: F = 2.33, p = 0.002, q = 0.012) (Supplemental Fig. S2a and Supplemental Fig. S2b). Another sub-analysis showed the house dust microbial composition was approaching but not significantly different in houses that had an asthmatic vs those that did not have an asthmatic present in the home (Supplemental Fig. S2c) (p=0.135).We trained a random forest classifier to differentiate the neighborhoods using the dust microbiome compositions, and the classifier achieved an overall accuracy of 0.78, which was 2.8 times better than baseline accuracy (assigning the most common category to all samples). The model predicted NUS accurately 75% of the time; TUS 60% of the time: high SES NMX 75% of the time; and low SES NMX 100% of the time (Fig. 1). When the model predicted the wrong neighborhood, it predicted the correct country. Our random forest model and ANCOM for comparing neighborhoods both found two genera that were enriched in the dust microbiome of both the low SES NMX and high SES NMX groups (*Aeromonas, and Dysgonomonas)* and one genus and family that was enriched in just the dust microbiome samples from the low SES NMX (*Ornithinimicrobium* and *Intrasporangiaceae,* respectively) (Supplemental Fig. S3).

The PICRUSt analysis predicted that there were 68 significantly differentially abundant functional pathways in the house dust microbial communities present when comparing the US to MX (Supplemental Fig. S4). Twenty-two significantly different functional pathways of the microbial communities in the dust existed between high SES NMX vs low SES NMX vs NUS, as compared to TUS (Supplemental Fig. S5). The PICRUSt analysis predicts that the microbes present in the house dust in low SES NMX had super pathway of polyaminine biosynthesis III capabilities. When comparing the low SES NMX to the high SES NMX, the microbes present in the house dust in the low SES NMX neighborhood had greater pyrimidine deoxy ribonucleotises biosynthetic, benzoyl CoA anaerobic degradation, nitrate reduction, and spirilloxanthin and 2.2-diketo-spirilloxanthin biosynthetic capabilities (Supplemental Fig. S5).

## Discussion

This study examined differences in household characteristics, environmental factors, socio-economic factors, and microbial composition of dust from households in neighborhoods across the US-MX Border, in which we previously documented differential rates of asthma prevalence [29]. We identified multiple structural, environmental, and human factor differences between the US and MX homes, which may have led to the differences seen in dust loading and microbial diversity across the dust samples collected from the homes. The bacterial genera *A. Alishewanella* and *Chryseomicrobium* were found to be enriched in the dust from homes within MX, while *Geodermatophilus* was found to be enriched in the dust from homes within the US.

This study found multiple housing characteristics that differed between MX and the US, differences that may contribute to the higher prevalence of asthma in children of Mexican descent living in the US compared to MX [29]. Homes in the US were more likely to be on paved roads and to have flushing toilets, piped/municipal water, more rooms and bathrooms, and evaporative cooling and/or air conditioning compared to MX homes. Although only 26.3% of individuals in the US drank their tap water, this was significantly higher than in MX (2.6%). It has been shown that municipal water has fewer commensal microbes present and that drinking of municipal water is associated with higher prevalence of asthma and allergies [21, 30]. Floors were more likely to be carpeted in the rooms where the dust was collected in the US. Carpets in the home may be an important risk for asthma development as they can be a reservoir for mildew, mold, allergens, and chemical hazards (e.g., pesticides, metals, flame retardants, per-and polyfluoroalkyl substances (PFAS), and can contribute to poor indoor air quality [31, 32]. Although carpets typically have greater dust loading than hard floors, the homes in MX had significantly greater dust loading than the ones in the US, despite the lack of carpet (Tables 2 and 3). This suggests that differences in the household density (particularly the number of children), the presence of pets, and other household structural factors between these communities might account for the greater dust loading in MX.

Microbial populations in indoor environments, where we live and eat, play an important role in human health. Environmental dust exposure early in life appears to influence what bacteria colonize the gut, skin, and nasal microbiome [33–36]. It has been proposed that part of the reason for increasing asthma and allergy prevalence worldwide is due to shifts in our lifestyles towards more Western or Modernized ways of living, which has led to a decrease in microbial exposure during a critical period of immune development [37]. Individuals with exposure to more diverse bacteria have lower rates of allergic diseases [38, 39]. We found significant differences in the microbiome composition of dust collected from homes in MX compared to the US (Fig. 1). Previous studies have shown a link between house dust microbial composition and risk of allergic asthma development. Children raised in Amish communities have lower rates of allergic sensitization and asthma than those from Hutterite communities. Stein et al demonstrated that house dust from an Amish community had a different microbial composition than the house dust from a Hutterite community. Further, mice that received the dust from the Hutterite houses intranasally had decreased airway reactivity and eosinophilia, which are markers of allergic asthma [22]. There was no difference in microbial dust composition between homes with and without an occupant that reports having asthma. There are likely multiple reasons for this finding. First, the study was not powered to find a difference based on asthma prevalence, and the study simply asked if a person in the household had asthma and not whether they grew up in that house. Also, the study did not use a validated tool to screen for asthma in all household occupants, so there may be a difference in asthma detection between the cities.

House dust microbial composition is affected by outdoor and indoor environments, including structural characteristics, as well as household occupants and their activities in the home. Any combination of the significant differences between the homes in MX and the US may have led to the differences observed in the microbial composition of the house dust. A sub-analysis showed that there was a difference in beta diversity (unweighted UniFrac) of the dust microbiome when comparing the homes by road type and presence of air conditioning. Using an air conditioner changes the indoor environment by changing the temperature and humidity, which would lead to differences in microbial growth indoors [40], as well as through filtering of the air. Homes with air conditioners are less likely to have windows open, and therefore less dust is likely to blow into the home. Furthermore, it has been shown that as regions become more industrialized and homes are constructed in a manner where they are more tightly sealed, the microbiome diversity decreases [41]. The structural differences and occupant behavioral differences between US and MX are likely to have led to the differences in microbial diversity in the household dust.

We have previously shown that these two regions have different prevalence of childhood asthma, and this study also found differences in house dust composition between the two regions. The dust from the US homes was more enriched with the genus *Geodermatophilus*, whereas the dust from Mexican homes was more enriched with *A Alishewanella* and *Chryseomicrobium*. While the genera of bacteria found in house dust in both the US and MX have not been previously identified as related to asthma prevalence, *Ornithinimicrobium* (more abundant in the low SES NMX neighborhood) and *Geodermatophilus* are part of the phylum Actinobacteria, which has been shown to be both negatively and positively associated with allergic disease [25, 42–46]. To our knowledge, *Geodermatophilus* has not been specifically linked to being a risk for asthma development previously, but its presence in school dust has been linked to “Sick Building Syndrome” [47]. The genus *Alishwaneela* is a gram-negative species that have endotoxin present in their cell walls. Previous studies have shown that higher levels of endotoxin in a child’s environment are related to lower incidence of atopic asthma and allergic diseases [22, 48, 49]. *Alishwenella* is also in the phylum of *Proteobacteria*, which, much like Actinobacteria, has been shown to be both negatively and positively associated with allergic disease in different studies [42, 43, 46, 50]. *Chryseomicrobium* is in the family *Planococcaceae.* In a study by Ta et al. it was shown that *Pamnomicrobium*, which is also in the family Planococcaceae, was protective against developing allergic eczema [44]. The inconsistencies in the direction of these relationships may be explained by the need to classify beyond the phylum level, which is very broad, or the presence of critical time points at which exposure protects against onset of asthma but may be harmful once asthma has developed. In any case, the differences found in microbial exposure between the sites suggest promising areas for future research related to asthma in the Border region.

Although some microbes in the dust are unlikely to be active metabolically, others are living and metabolically active, and so may play a critical role in alteration of host microbiome. They can colonize the host, where they can become metabolically active and play a key role in risk of asthma development. A PICRUSt analysis was done to identify potentially relevant metabolic pathways present in the house dust from homes located in MX vs US. There were 23 biosynthetic pathways, 38 degradation/utilization/assimilation pathways, and 7 pathways involved in generation of precursor metabolites that were higher in the house dust from MX. Some of the pathways found to be more prevalent in the house dust from MX are involved in biosynthesis of molecules that are known to be protective against asthma (Supplemental Fig. S4), such as short chain fatty acids and pyrimidine [51, 52]. There have been multiple studies that show the importance of metabolites produced by the microbiota that colonize humans in protecting against asthma via alterations in the epithelial barrier function and immune system regulations. Many of the possibly up-regulated functional pathways present in the house dust from MX have been examined in other studies and have been shown to be protective against asthma or helpful in asthma control. For example, the UDP − N−acetyl − D−glucosamine and the mycolate biosynthetic pathways that were higher in the house dust from MX have been shown to down-regulate allergic airway inflammation [53–56] (Supplemental Fig. S4).

Limitations of this study include that collection of the house dust microbiome samples occurred at a single time-point and during a single season. There was significant differences in the ambient temperatures during sample collection across the border and this was likely related to more of the MX samples being collected in the spring/summer months and more of the US sample being collected in the winter. Future studies would benefit by looking at multiple time points to assess the variability of household microbiomes. The high SES NMX homes were all part of a subdivision with identical floor plans, which could drive some of the differences observed between the low and high SES neighborhoods in NMX. We used 16S rRNA amplicon sequencing to characterize the house dust, which generally supports taxonomic resolution only at approximately the genus level, although species level differences may affect human health. Shallow or deep shotgun metagenomics, although more expensive, would improve microbial identification, and deep shotgun metagenomics would provide more accurate functional profiles of the samples. PICRUSt uses 16S rRNA data to extrapolate metagenome composition and provides relatively lower confidence functional pathway profiles of samples. Given that asthma is a common reason for presentation to outpatient clinics and household recruitment was through outpatient clinics, there may have been selection bias because families that had an asthmatic child in the home may have been more likely to enroll in the study. However, this bias could have been represented on both sides of the Border. We did not investigate differences in pollutants in the collected dust, such as metals or pesticides, which could affect the dust composition and microbial diversity, along with the growth of opportunistic bacterial pathogens [57].

In conclusion, despite TUS, NUS, and NMX being geographically close (< 100 km) and having similar climates, homes across this Border region differ in ways that lead to significantly different indoor environments. Mexican and US households differed in years of education; household income; the percentage of homes that had paved roads, flushing toilets, and piped water; the number rooms and bathrooms present in the home; and presence and type of cooling and flooring. Some of these household differences may have led to the significant differences we observed in the microbial composition of the house dust collected from MX or US homes. The dust from the US homes was more enriched with the genus *Geodermatophilus*, whereas the dust from Mexican homes was enriched with *A. alishewanella* and *Chryseomicrobium*. Future research should assess whether exposure to these bacteria during critical windows in early life may offer protection from development of asthma or allergic disease.

## Methods

### Study Population

Patients at each of three clinics were approached from September to December of 2016 to participate in this study: El Rio Community Health Center in TUS (n = 20), Mariposa Community Health Center in NUS (n = 20), and the Secretaría de Salud de Sonora in NMX (n = 40). In NMX, 20 households were recruited from a traditionally high SES neighborhood (high SES) and 20 from a traditionally low SES neighborhood (low SES). All of the households that were recruited from the high SES neighborhood in NMX were in a subdivision neighborhood where the houses were constructed with identical layouts. Families were eligible to participate in this study if at least one parent was of Mexican descent and had at least one child younger than 5 years old. The University of Arizona Human Subjects Protection Program approved all study materials (IRB approval number: 1607687201), in addition to all necessary permissions and reviews from the US/Mexico Border Commission, the Secretaría de Salud de Sonora, Mariposa Community Health Center and El Rio Community Health Center.

### Questionnaire

During the home visit, a questionnaire was administered orally in English or Spanish by a trained research assistant to obtain information on household demographics, asthma prevalence, sanitation measures, drinking water sources, and pets in the home. Multiple household characteristics were assessed (e.g., mildew, water damage, structural characteristics, and type and number of bathrooms).

### Dust Sample Collection

House dust was collected using a Hoover CH3000 vacuum cleaner equipped with a pre-weighed sterilized X-Cell 100 dust collection sock (Midwestern Filtration, Cincinnati, OH) inserted in the crevice tool. To collect floor dust, a one-meter square template was laid on the floor in the child’s room and vacuumed for five minutes. If a child did not have their own room, then the sample was collected in the room where the child regularly spends most of their time. The sock filter holding the collected dust was placed in a plastic bag sterilized under UV light in a hood. The vacuum and its accessories were cleaned with disinfectant wipes and sprayed with isopropyl alcohol between sampling each house. Collected dust samples were transported in a cooler with ice packs to the laboratory in the Medical Research Building at the University of Arizona in Tucson, AZ.

In the laboratory, each dust sample was transferred from the filter sock to a pre-weighted 50 ml sterilized centrifuged tube. The centrifuge tube with the dust sample was weighed three times using a Mettler Toledo AB54 Precision Balance Weight Scale (Mettler Toledo International, Inc., Columbus, OH). The average of three measurements was then recorded. All environmental samples were stored in a −80 C freezer until analyzed. Frozen dust samples were shipped to the Pathogen and Microbiome Institute at Northern Arizona University for amplicon library preparation and sequencing.

### DNA Extraction

DNA was extracted using the MoBio Powersoil DNA isolation kit (Qiagen) with an additional mechanical lysis. Briefly, samples were placed in a lysing matrix E tube (MP Biomedical) with 600 µl of Buffer RLT Plus and lysed in 30 second increments for a total of 6 minutes at 10,000 x g. Samples were sat resting for 30 seconds between each bead beating to prevent heating. Extraction continued following the manufacturer’s protocol. DNA was quantified using a NanoDrop 2000. Extraction blanks, which did not contain any sample during the extraction, were carried throughout the entire extraction and 16S rRNA gene sequencing.

### 16S rRNA gene sequencing

Sample processing and sequencing were performed using the Earth Microbiome Project (www.earthmicrobiome.org) protocols. The barcoded primers 515F/806R were used to target the V4 region of the 16S rRNA gene, as previously described [58]. Each PCR reaction contained 2.5 µl of PCR buffer (TaKaRa, 10x concentration, 1x final), 1 µl of the Golay barcode tagged forward primer (10 µM concentration, 0.4 µM final), 1 µl of bovine serum albumin (Thermofisher, 20 mg/mL concentration, 0.56 mg/µl final), 2 µl of dNTP mix (TaKaRa, 2.5 mM concentration, 200 µM final), 0.125 µl of HotStart ExTaq (TaKaRa, 5 U/µl, 0.625 U/µl final), 1 µL reverse primer (10 µM concentration, 0.4µM final). All PCR reactions were filled to a total 25 µL with PCR grade water (Sigma-Aldrich) then placed on a ThermalCycler. ThermalCycler conditions were as follows: 98°C denaturing step for 2 minutes, 30 cycles of 98°C for 20 seconds, 50°C for 30 seconds, 72°C for 45 seconds, and a final step of 72°C for 10 minutes. PCR was performed in triplicate for each sample and an additional negative control was included for each barcoded primer. A post-PCR quality control step was performed using a 2% agarose gel (ThermoFisher). Extraction blank controls were processed through the 16S PCR with the same methods as samples. Barcode primer NTCs controls were carried through the agarose gel step. If amplification was present for negative controls, the PCR was repeated with a new barcoded 806R primer. Following agarose gel, PCR product was quantified using the Qubit dsDNA High Sensitivity Kit (ThermoFisher) and the Qubit fluorometer 4. PCR products were pooled at equimolar concentrations of 50 ng. Quality of the pool was assessed with the Bioanalyzer DNA 1000 chip (Agilent Technologies), combined with 1% PhiX and sequenced on the Illumina MiSeq using the 600-cycle MiSeq Reagent Kit VX (Illumina).

### Data analysis

#### Demographic and housing characteristic analysis

The house and family characteristics were analyzed by country (US, MX) and by neighborhood (TUS, NUS, high SES NMX, low SES NMX). Data analysis was conducted with Stata v16. (StataCorp, College Station, TX). Comparisons between the two countries were assessed with Fisher’s Exact and Mann Whitney U tests. Comparisons between the neighborhoods were made using Fisher’s Exact and Kruskal-Wallis tests. Non-parametric tests were used, as the data had a skewed distribution. An alpha level of 0.05 was considered statistically significant.

##### Microbial Data Analysis:

The microbiome sequencing data was analyzed using QIIME 2 2021.2 [45]. The emp-paired (Hamady & Knight 2009) action in the q2-demux plugin was used to demultiplex the data. We used the denoise-paired action in the q2-dada2 [59] plugin to perform sequence quality control and define amplicon sequence variants (ASVs) with the following parameter settings: trim-left-f = 0; trim-left-r = 0; trunc-len-f = 200; trunc-len-r = 230. A phylogenetic tree was created using align-to-tree-mafft-fasttree [60, 61] in q2-phylogeny, for use with phylogenetic alpha and beta diversity metrics. q2-diversity’s core-metrics-phylogenetic action was used to compute Faith’s Phylogenetic Diversity Index [62], Unweighted Unifrac [63] and Weighted Unifrac [64] at an even sampling depth of 23,717. The alpha-rarefaction [65] action in the q2-diversity plugin was used to generate rarefaction curves based on Faith’s Phylogenetic Diversity Index [62] to confirm that the richness of the samples was stable around the chosen sampling depth. The beta-group-significance action in the q2-diversity plugin was used to run PERMANOVA pseudo-F tests on across sample groupings of interest. False-discovery-rate (FDR) correction was applied to correct for multiple comparisons. These FDR-corrected p-values will be presented as “q-values” in this text. Taxonomic annotation of ASVs was performed using the qiime feature-classifier [66, 67] classify-sklearn method using the SILVA 138 classifier [68]. A taxonomic bar plot was generated for the data based on the SILVA-based taxonomy. An ANCOM analysis was applied to identify differentially abundant taxa at the genus level across cities and countries using qiime composition ancom [69]. Finally, a random forest model was built using the qiime sample-classifier [66, 67] classify-samples method to predict the country and neighborhood of origin of the microbiome sample. This model was trained on the ASV table combined with the phylum and genus tables that were generated by collapsing ASV into taxa using the collapse action in the q2-taxa plugin. To predict the abundance of gene families and related functional pathways of microbial communities present in the house dust, a Phylogenetic Investigation of Communities by Reconstruction of Unobserved States (PICRUSt) analysis was completed, which is used to predict metabolic pathways based on 16S rRNA results. The significant differences between the functional pathways present in the US vs MX or the different neighborhoods’ dust was analyzed using MaSaLin2 software [70]. TUS was used as reference when comparing the functional pathways in the dust between the communities (i.e., high SES NMX, low SES NMX and NUS).

## Supplementary Material

Supplement 1

## Figures and Tables

**Table 3.** Floor Dust loading (mg/m^2^) in relation to household characteristics. MX=Mexico; US= United States of America Statistical Test: Mann Whitney U Test.

Variable	N	Geomean	GSD	P-value
**Location**				0.04
US	40	107.4	2.19	
MX	40	172.4	2.93	

**Self-Reported asthma**				
No one with asthma	66	137.7	2.79	0.89
At least one person with asthma	14	128.9	1.89	

**Nearest road type**				0.02
Dirt	21	285.9	3.03	
Asphalt/Tile	59	106.3	2.18	

**Number of rooms**				0.04
2 or less bedroom	38	166.7	2.76	
More than 2 bedrooms	42	113.3	2.44	

**Pets**				
No pets	50	115.3	2.15	0.01
Owning at least one pet	30	176.9	3.27	

**Number of adults**				
2 or less adults	47	130.9	2.11	0.87
More than 2 adults	33	143.8	3.40	

**Number of children**				
2 or less children	54	115.3	2.27	0.01
More than 2 children	26	192.1	3.19	

**Total number of residents**				
4 or less residents	40	105.1	2.16	0.01
More than 4 residents	40	176.4	2.94	
