# Supplementary material for: Environmental and Structural Factors Associated with Bacterial Diversity in Household Dust Across the Arizona-Sonora Border
Source: Res Sq. 2023 Sep 29:rs.3.rs-3325336. Preprint. [Version 1] doi: 10.21203/rs.3.rs-3325336/v1 (PMC10571632; doi:10.21203/rs.3.rs-3325336/v1)
Supplement: Supplement 1 [file NIHPPRS3325336V1-supplement-1.pdf]

Supplementary Files

This is a list of supplementary files associated with this preprint. Click to download.

- DustmicrobiomeUSMXsuplemental.docx
- DustmicrobiomeUSMXTable1.docx
- DustmicrobiomeUSMXtables2.docx
